# Therapeutic Approaches of Resveratrol on Endometriosis via Anti-Inflammatory and Anti-Angiogenic Pathways

**DOI:** 10.3390/molecules24040667

**Published:** 2019-02-13

**Authors:** Ana-Maria Dull, Marius Alexandru Moga, Oana Gabriela Dimienescu, Gabriela Sechel, Victoria Burtea, Costin Vlad Anastasiu

**Affiliations:** Department of Medical and Surgical Specialties, Faculty of Medicine, Transilvania University of Brasov, 500019 Brasov, Romania; dullana2005@yahoo.com (A.-M.D.); moga.og@gmail.com (M.A.M.); victoriaburtea@yahoo.com (V.B.); canastasiu@gmail.com (C.V.A.)

**Keywords:** cytokines, resveratrol, endometriosis, anti-inflammatory, inflammatory disease

## Abstract

Endometriosis represents a severe gynecological pathology, defined by implantation of endometrial glands and stroma outside the uterine cavity. This pathology affects almost 15% of women during reproductive age and has a wide range of consequences. In affected women, infertility has a 30% rate of prevalence and endometriosis implants increase the risk of ovarian cancer. Despite long periods of studies and investigations, the etiology and pathogenesis of this disease still remain not fully understood. Initially, endometriosis was related to retrograde menstruation, but new theories have been launched, suggesting that chronic inflammation can influence the development of endometriosis because inflammatory mediators have been identified elevated in patients with endometriosis, specifically in the peritoneal fluid. The importance of dietary phytochemicals and their effect on different inflammatory diseases have been highlighted, and nowadays more and more studies are focused on the analysis of nutraceuticals. Resveratrol is a phytoestrogen, a natural polyphenolic compound with antiproliferative and anti-inflammatory actions, found in many dietary sources such as grapes, wine, peanuts, soy, berries, and stilbenes. Resveratrol possesses a significant anti-inflammatory effect via inhibition of prostaglandin synthesis and it has been proved that resveratrol can exhibit apoptosis-inducing activities. From the studies reviewed in this paper, it is clear that the anti-inflammatory effect of this natural compound can contribute to the prevention of endometriosis, this phenolic compound now being considered a new innovative drug in the prevention and treatment of this disease.

## 1. Introduction

Endometriosis represents a severe gynecological disease that affects almost 15% of women in reproductive age, described by the implantation of endometrial tissue outside the uterus [1]. In endometriosis, endometrial tissue fragments are present mainly on the ovaries, on the pelvic peritoneum, in the pouch of Douglas, and the rectovaginal septum [2]. Frequently, this dissemination of the endometrial cells is explained by the theory of retrograde menstruation, consisting of the presence of menstrual blood in the abdominal cavity, due to reflux through the Fallopian tubes, an approach first described by Sampson in 1927 [3]. The favorable hormonal environment and some immunological factors are involved in the implantation of endometrial cells in abnormal sites outside the uterus and in the failure to eliminate these cells from the inappropriate places [4].

There are three types of endometriosis, according to its localization in the pelvis: peritoneal, ovarian, and rectovaginal, presented in the first stage of implantations as red lesions similar to the eutopic endometrium. In time, these red lesions become black by an inflammatory reaction that provokes a process of scarification [2].

The clinical presentation of endometriosis is variable and includes some severe symptoms: dyspareunia, chronic pelvic pain, dysmenorrhea, and subfertility or infertility [5]. The severity of symptoms increases with age [6].

Even the pathogenesis and the mechanisms of initial development and subsequent progression of endometriosis are still unclear. There are proofs that this disorder is a pelvic inflammatory process and chronic inflammation has a significant role in the development and progression of the pathology [7].

Based on this pro-inflammatory hypothesis, various studies report that in the peritoneal fluid of patients with endometriosis increased numbers of activated macrophages, cytokines, angiogenic factors, and growth factors have been identified, [8] produced through the alteration of the regular activity of peritoneal cells [9].

A study conducted by Kobayashi et al. [10] highlights that endometriosis may be stimulated through the activation of the inflammatory cells. Also, subsequent inflammation and microbial infections of the upper genital tract are also involved in the initial development and progression of the lesions [10]. Peritoneal oxidative stress is also considered a significant component of endometriosis-related inflammation, and it can regulate genes that encode immunoregulators, cytokines, and cell adhesion molecules [11].

In the literature, there have been several studies conducted to highlight the importance of diet and its impact on the prevention and treatment of a wide range of diseases, raising more and more interest in the analysis of dietary polyphenols [12]. Polyphenols are micronutrients found in dietary sources and proof of their impact on the prevention of diseases is emerging [13]. The most common coccurrences of polyphenols are in herbs, fruits, beverages, vegetables and spices, several of these polyphenols have been proven to exhibit anti-inflammatory actions. Besides, several studies maintain that the consumption of food abundant in polyphenols can reduce the incidence of chronic inflammatory pathologies [14].

Resveratrol is a natural phytoalexin (trans-3,5,40-trihydroxystilbene), synthesized by plants due to ultraviolet radiation and fungal infections [15,16]. High levels were identified in grapes, wine, berries, Itadori tea, nuts, and stilbenes. Analysis of peanuts, raisins, and Itadori tea confirm the predominance of trans-resveratrol glucoside and, in counterpoint, the study of red wine shows that it contains mainly the aglycones cis- and trans-resveratrol forms [17].

Several studies indicate that resveratrol possesses various beneficial actions, including anti-neoplastic, anti-inflammatory, anti-oxidative, anti-microbial, anti-atherogenic, and anti-angiogenic properties [16,18]. It is also useful because it may provide cardiovascular protection [19].

Regarding the mechanism of action of resveratrol, numerous studies have shown that it includes multiple cellular targets affecting signal transduction pathways. AKT (protein kinase B) represents these pathways, Signal transducer and activator of transcription 3 (STAT3), ribosomal protein S6 kinase beta-2 (RPS6KB2), mitogen-activated protein kinase 1/3 (MAPK1/3; ERK1/2), Mitogen-Activated Protein Kinase 14 (MAPK14 (p38)), protein kinase C, and peroxisome proliferator-activated receptors (PPAR) gamma [20,21,22]. Some of the pathways are relevant for the mechanisms of endometriosis-related development on the impact of resveratrol as an anti-inflammatory agent [23,24]. This hypothesis supports that resveratrol seems to be a possible innovative alternative agent in the prevention and treatment of severe disease, but further studies are necessary to elucidate the useful potential of this phytochemical and also the potential adverse effects that may appear.

Bruner-Tran et al. [25] pointed out the inhibition of endometriotic lesions by resveratrol in a study published in 2011. Their study concluded that the oral gavage of resveratrol could reduce the quantity and dimensions of endometriotic lesions. The in vivo study was performed on nude mice and consisted of transplantation of induced human endometrial tissue into the peritoneal cavity of these mice. This effect of resveratrol was linked to reduced proliferative action and up-regulation of apoptotic cell death into the lesions.

The essential mechanism of resveratrol in the prevention of endometriosis is considered anti-inflammatory activity. It has been demonstrated to be manifested through the inhibition of prostaglandin synthesis via the inhibition of COX enzyme synthesis, inhibition of activated immune cells, and inhibition of pro-inflammatory cytokines [26].

Endometriosis, a chronic inflammatory disease is a frequent gynecological pathology, which has a severe impact on women worldwide, therefore by understanding the pathophysiology, and the mechanism of action of resveratrol we may improve the development of this disorder and the treatment strategies, using natural products. With low toxicity, high accessibility, and low price, resveratrol may become an alternative therapeutical agent for the prevention and treatment of endometriosis.

## 2. Biochemistry of Resveratrol

Resveratrol is a phytochemical found in high concentration in grapes, wine, tea, peanuts, and berries also called a “miracle molecule” because it exhibits many beneficial properties [27,28]. This molecule was discovered and described for the first time in the year 1939 [29,30] when it was isolated from the roots of *Veratrum grandifloorum* and in the year 1963, it was isolated from a plant used in complementary medicine in China, *Polygonum cuspidatum*. Since the first discovery of the compound, numerous scientific researches have been conducted to study the activity of resveratrol. In 2002, Burns et al. [31] reported 92 new resveratrol compounds from the *Dipterocarpaceae*, *Vitaceae*, *Paeoniaceae*, *Gnetaceae*, *Leguminosae*, *Polygonaceae*, *Gramineae*, *Cyperaceae,* and *Poaceae* families. According to Sobolev et al., two new dimers of resveratrol were isolated from peanut seeds [32], and this phytochemical has also been separated from baking chocolate, cocoa powder, and dark chocolate (0.35–1.85 mg/kg in commonly consumed quantities) [33]

Resveratrol is part of the large chemical class of stilbenes and as well as the resveratrol molecule and its analogs, a stilbene is considered a monomer, a primary building block that leads to subsequent polymerization. In the last five years, over 60 naturally occurring stilbenes have been isolated, and it is a fact that stilbenes exhibit a large wide of oligomeric constructions and polymerization [34].

**Figure 1:**
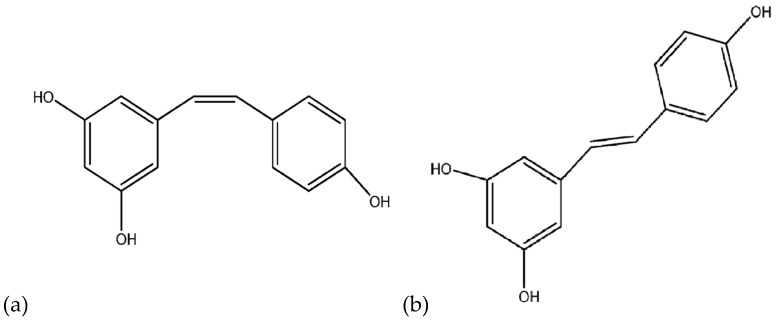
The two isomers of resveratrol; (**a**) cis-resveratrol and (**b**) trans-resveratrol [37].

The universal skeleton of stilbenes is a C6–C2–C6 unit, namely, a 1,2-diphenylethylene moiety [34]. Resveratrol is a polyphenolic phytoalexin that possesses two aromatic rings linked to each other by a double ethylene bridge [35]. The chemical structure of resveratrol (trans-3,5,4′-trihydroxystilbene) is responsible for the two isomeric forms, *cis*-resveratrol, and respectively *trans*-resveratrol [36]. In plants, wine, and in natural food, resveratrol is found as both *cis*- and *trans*-isomers, although the significant and more stable form of resveratrol is *trans*-resveratrol, which is useful as a preventive agent in cancer, vascular diseases, infections, etc. [37]. Figure 1 illustrates the two isomers of resveratrol.

**Figure 2:**
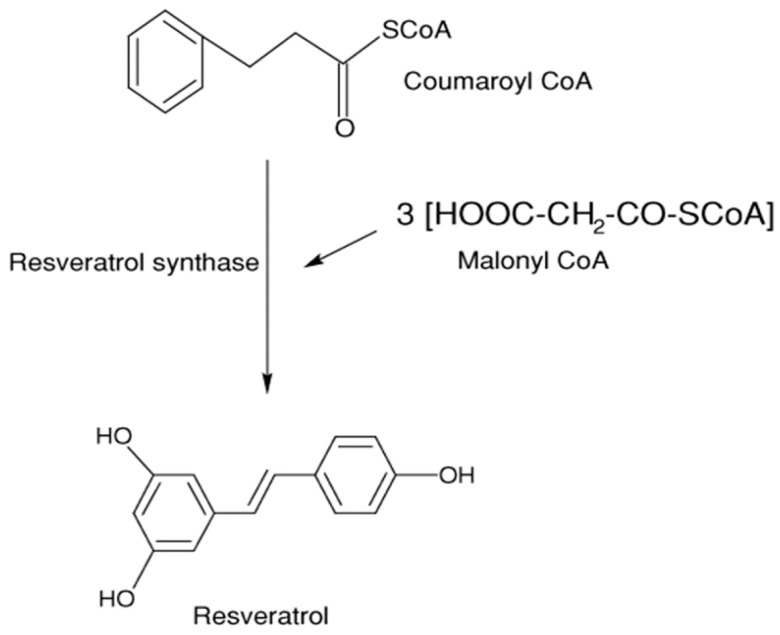
Resveratrol synthesis from malonyl-CoA and 4-coumaroyl CoA [43].

The synthesis of resveratrol is a condensation reaction including three molecules of malonyl-CoA and one molecule of 4-coumaroyl CoA. From this reaction, out of resveratrol, also result four molecules of CO_2_ [38]. The synthesis of resveratrol is shown in Figure 2.

The conversion of *cis*-isoform into *trans*-isoform is called cis-isomerization, and this phenomenon is possible when a *trans*-isoform is exposed to UV radiation or sunlight [39,40]. Therefore, because the *cis*-form is less stable [39], many studies use *trans*-resveratrol for administration, to highlight the biological effects of this phytoalexin.

As with any others polyphenols, resveratrol can undergo an auto-oxidation process and O_2_ and H_2_O_2_ are produced. This can also result in a mixture of quinines and semiquinones that may be cytotoxic [41]. Regarding the metabolism of resveratrol, studies revealed that it is metabolized into glucuronide and sulfate conjugates. Regarding the crystalline resveratrol and its glucoside, a study of Jensen et al. [42] maintains that both of these forms are stable for more than three months, a low degree of degradation may occur in various conditions such as air exposure, fluorescent light, UV light, high temperature, etc.

Recently, five tetramers of resveratrol were separated from the roots of *Vitis amurensis*: amurensins I–M, together with five resveratrol tetramers, named isohopeaphenol, (+)-vitisifuran A, heyneanol A, (+)-hopeaphenol, vitisin A, From all of these tetramers, vitisin A, (+)-hopeaphenol, isohopeaphenol, heyneanol A, and (+)-vitisifuran A showed a potent anti-inflammatory activity and also presented strong inhibition of the biogenesis of leukotriene B4 (LTB4) [44].

## 3. Development of the Endometriosis-Role of Inflammation

Endometriosis is characterized by functional endometrial stroma and gland implants outside of the uterine cavity. The main symptoms of this gynecological disease are chronic abdominal pain, dysmenorrhea, infertility [45,46], and anxiety or depression related to the severity of the pelvic-abdominal pain [47].

Regarding the pathophysiology of endometriosis and the biological mechanism of endometriosis-associated pain, these still remain controversial and unclear. Endometriosis is considered a multi-factorial condition with immunological, genetic, and hormonal environment contribution, characterized by an abnormal expression of inflammatory factors [48]. An essential step in the progression of endometriosis is represented by the link between inflammation and activation of the aromatase gene in the endometrium, followed by the local production of estrogens [49]. In healthy patients, the aromatase gene is inhibited, but in affected women, the promoter of this gene is activated by exposure to pro-inflammatory prostaglandins (PGE2). A malicious cycle between estrogen production and chronic inflammation is created, and through this cycle, the survival of heterotopic endometrial cells is maintained [50]. NF-kB is the link between aromatase expression and inflammation in endometriosis, the activation and translocation of NF-kB from the cytoplasm to cell nuclei being the first step to induce the inflammation process [51].

In patients with endometriosis and adenomyosis, the nuclear factor-kB subunit bound to a cell has been observed more often than in control cases [52,53]. The inflammatory environment in endometriosis points out an increased production of estrogens, which also increases prostaglandin production through NF-kB and COX-2 activation [54]. Recent studies also suggest that an essential cause of endometriosis development is neurovascular formation, or angiogenesis [55]. Angiogenesis is a complex process of new blood vessels formation that involves the extravasation of growth factors, degradation of the extracellular matrix, and new tube formation by endothelial cells. Endometriosis is dependent on the development of new blood vessels [56,57] that also associate a wide range of angiogenesis-related factors, such as VEGFR, VEGF, Delta-like 4 (Dll4)-Notch signal pathways, and angiopoietin [58].

VEGF and VEGFR are angiogenesis-related factors that affect the proliferation, migration, and permeability of the cells. A study of Gagne et al. [59] showed that the level of vascular endothelial growth factor in the peritoneal fluid of type IV endometriosis patients is significantly higher compared to type I/II endometriosis. VEGFC is an angiogenesis factor that acts on endothelial cells and promotes angiogenic responses through VEGFR2-mediated pathways, to improve endothelial function and vascular permeability in endometriosis [60].

Other constituents implicated in the development and progression of this chronic disease, are matrix metalloproteinases (MMPs), cyclo-oxygenase (COX), tumor necrosis factor (TNF-α), and hypoxia-inducible factor 1α (HIF-1) [48]. MMPs are involved in endometrial adhesion and angiogenesis, TNF- α promotes angiogenesis, and COX promotes the implantation of heterotopic endometrial cells [61,62]. Ectopic implants of the endometrial cell have shown increased expression of cyclo-oxygenase-2, while cyclo-oxygenase-2 inhibitors have been largely studied in the treatment of endometriosis-related abdominal pain [63]. The activity of MMP-2 in endothelial cells is significantly increased by PGE2 and is suppressed by the inhibition of COX-2 and all those factors, either directly or indirectly, are able to affect endometriosis-associated angiogenesis [64].

In the inflammatory panel of endometriosis, NF-kB has a crucial role in the progression of the disease. This factor is activated by several cytokines pro-inflammatory: tumor necrosis factor - α, interleukin 1β, and NF-kB activates multiple inflammatory mediators like IL-8 [65]. Activated macrophages are the key in the defense against infections because they can secrete cytokines pro-inflammatory: interleukin -1, interleukin -6, interleukin -12 and tumor necrosis factor-α and uncontrolled activation of these factors results in persistent inflammation. In the context of chronic inflammation, high levels of these cytokines have been identified in the peritoneal fluid of patients with endometriotic lesions [66]. TNF-α promotes inflammation in the Fallopian tubes, and this results in tissue repair and fibrosis in the tubes, which impairs reproduction, leading to poor quality of oocytes. IL-1 can promote angiogenesis in endometrial lesions and interfere with peritoneal immune surveillance. IL-8 has been identified in high levels in the peritoneal fluid of the patients with endometriosis and promotes cell attachment and cell growth [67].

**Figure 3:**
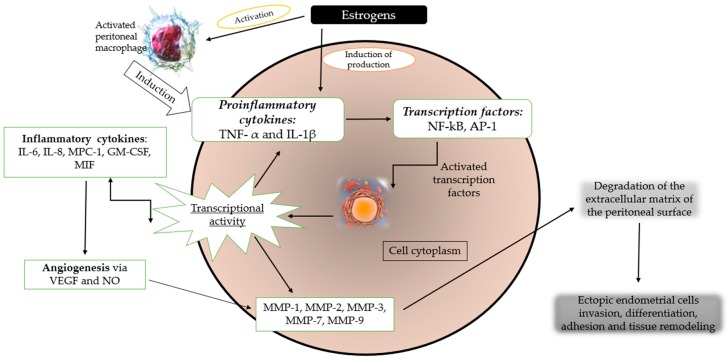
Illustration of molecular pathways in the development of endometriosis [7,68,73,74,75].

So, the basis of endometriosis pathophysiology is represented by the pro-inflammatory cytokines axis, which was named by Soares et al. [68] “the crossroads of the molecular pathways.” This process involves cytokines (tumor necrosis factor-α, interleukin-6, interleukin-8, monocyte chemoattractant protein, macrophage inhibitory factor, and granulocyte macrophage colony-stimulating factor) [69]. Matrix metalloproteinase-1, matrix metalloproteinase-2, matrix metalloproteinase-3, matrix metalloproteinase-7, matrix metalloproteinase-9 [70], nitric oxide (NO), and VEGF are involved in neoangiogenesis [71]. Figure 3 illustrates the pattern of the most relevant molecular pathways implicated in the pathophysiology of this pathology.

The purpose of the management of this chronic inflammatory disease is to improve chronic abdominal pain and successfully achieve a pregnancy in infertile women while the treatment is both medical and surgical. Regarding medical treatment, this involves a wide range of therapeutic agents including cyclo-oxygenase-2 inhibitors, TNF-α blockers, nuclear factor-kB inhibitors, statins, mitogen-activated protein kinase inhibitors, immunomodulators, MMP inhibitors, Metformin, antiangiogenic agents, and antioxidants [68].

Recent studies have shown the importance of natural therapy assessment for endometriosis treatment. Nowadays, natural compounds from food and various plants, named phytochemicals, are considered useful for the treatment of several diseases, including endometriosis. These new agents promise a new and revolutionary perspective in the treatment of endometriosis.

Resveratrol, the miraculous phytoalexin contained in grapes and red wine is an agent with multiple beneficial activities, and its role in endometriosis development and progression has been studied to demonstrate its effectiveness as a therapeutic agent. This study focuses on complementary medicines for the treatment of endometriosis, especially on the effect of resveratrol and examines its therapeutic efficacy and mechanism of action.

## 4. Anti-Inflammatory Molecular Mechanisms of Resveratrol

Resveratrol exerts different effects on various molecular pathways involved in inflammation, such as arachidonic acid, Nf-kB, Ah receptor or AP-1. We summarize in this subchapter how this natural compound affects these signaling pathways.

### 4.1. Arachidonic Acid Pathway

Various stimuli, such as hormones, cytokines, and stress signals activate the arachidonic acid (AA) pathway, under the action of phospholipase A2, the results being the release of this acid from the cell membranes [72]. Through the activity of lipoxygenase and COX, arachidonic acid is converted to several eicosanoids. Cyclo-oxygenase plays an essential role in inflammation because it catalyzes the formation of prostaglandin H2 (PGH2). The primary mechanism, namely the conversion of AA to PGH2 involves two steps: bisdioxygenation of arachidonic acid to PGG2 and peroxidative cleavage of PGG2 to PGH2, releasing the active biological prostanoids (PGE2, PGF2α, PGI2, and thromboxane A2) [72].

Regarding resveratrol, several types of research have pointed out that this compound interacts with the arachidonic acid pathway suppressing COX-2 effects on various levels. Subbaramaiah K et al. concluded in their study that the mechanism through which resveratrol inhibits inflammation is represented by suppression of PMA induced cyclooxygenase transcription in mammary epithelial cells, mainly through inhibition of the protein kinase C pathway [76]. This compound also prevents the induction of cyclooxygenase 2 promoter activity (known to be mediated by ERK-1 and c-Jun). Camp responses element (CRE) is also an essential element in the inflammatory process. When interfering with resveratrol, it suppresses COX-2 expression. Xie et al. [77] reported in their study that C-Jun (a component of activator protein 1) activates cyclooxygenase promoter through the camp responses element.

### 4.2. Aryl Hydrocarbon Receptor (AhR)

AhR has an essential role in the immune system, being a mediator of dioxin toxicity [78]. Furthermore, Esser et al. demonstrated in their study that aryl hydrocarbon receptor could bind different factors (estrogen receptors, NF- κB, E2F1) [79]. Because dioxin induces immunosuppression, agonists for AhR have been studied during the last years, aiming to identify natural products that interfere with this unique molecular pathway of inflammation. Resveratrol is one of the natural compounds that was identified to have antagonist effects on AhR [80,81]. It has been pointed out that essential roles in the regulation of the immune system are played by FoxP3+ Tregs and effector Th17 subset [82]. The research results of Bettelli E et al. showed that resveratrol inhibited the development of Th17 cells and FoxP3+ Treg [83]. Another in vitro study highlighted that resveratrol could block Th17 development [84]. These findings reflect the beneficial activity of resveratrol on inflammation, through inhibition of Th17.

### 4.3. Activator Protein 1 Pathway

In the inflammatory process the activator protein 1 is also involved, along with NF-κB, NFAT, and STATs, playing an essential role in the initiation of the process, by promoting the transcription of various biomolecules and pro-inflammatory cytokines. AP-1 factor includes multiple members of JUN, FOS, ATF, and MAF protein families [85]. Activation of AP-1 is induced by various cytokines (especially through JNK and MAPK signaling), hormones, growth factors (through extracellular-signal-regulated-kinase), and cellular stress. Cytokines activate this pathway especially through JNK and MAPK signaling. The cytokines (IL-2, IL-3, IL-4, IL-5, IL-13, IFN-γ, TNF-α) influenced by AP-1 are regulated by a transcription factor complex that involves NFAT. The activated AP-1 proteins have an essential role in the differentiation of T cells, it being known that several inflammatory diseases are characterized by this type of T-cell response [86,87]. Some studies suggested that resveratrol interferes with the inflammation process through AP-1 pathways, inhibiting COX-2 activity indirectly, after inhibition of AP-1. So, the AP-1 pathway is indispensable when discussing the anti-inflammatory effects of this natural compound [88,89].

### 4.4. NF-κB Pathway

The proteins of the NF-κB family proteins contain a Rel homology domain that serves as their dimerization, DNA-binding, and a primary regulatory domain, according to Ghosh et al. [90]. NF-κB proteins activation is dependent on the phosphorylation of IκB proteins. After the activation of IκB proteins, the released NF-κB proteins activate various target genes associated with cell proliferation, and inflammatory responses [91]. Manna et al. suggested that resveratrol modulates NF-κB through suppressing its activation in several cell types, blocking TNF-α and inducing activation of NF-κB [92]. Other researchers showed that this natural compound could prevent NF-κB activation by different stimuli besides TNF-α, such as other pro-inflammatory cytokines (IL-1β) or LPS, H2O2, okadaic acid, and ceramide [93].

## 5. Studies of Resveratrol as Anti-Inflammatory Agent in Endometriosis

Resveratrol, a natural non-flavonoid antioxidant, is a phytoalexin found in high levels in red wine, approximately 1.52 mg/L and in grapes skin, 50–100 µg/g [94]. Resveratrol has been shown to possess significant activity as anti-inflammatory, antioxidant, antiangiogenic agent and it also has immunomodulatory properties [95]. In demonstrating the anti-inflammatory effect of resveratrol several studies shown that this natural compound suppresses the production of ROS and inhibits COX-2 expression and prostaglandin synthesis [96]. In Table 1 and Table 2, we summarized the most representative preclinical and clinical studies conducted to demonstrate the efficiency of resveratrol in the management of endometriosis.

### 5.1. Preclinical Studies

In the initiation and progression of this disease, activation of peritoneal macrophages is a crucial step [97]. This mechanism is responsible for the increased lipid peroxidation. Several authors concluded that the increased production of free oxygen radicals, elevates oxidized lipoproteins in the peritoneal fluid and lower levels of SOD and GSH-Px are often identified in patients with endometriosis. Another marker of lipoprotein peroxidation is lysophosphatidylcholine, which was found elevated in patients with this pathology, according to Murphy et al. [98,99]. The imbalance oxidation–antioxidation appears to be responsible for the pathophysiology of endometriosis. The authors of this study identified increased levels of MDA (increased lipid peroxidation in peritoneal plasma and implants), but after supplementing with resveratrol in a dose-dependent manner, the increased levels were suppressed, speculating that this natural compound can have beneficial effects as a potent antioxidant [98,99,100,101,102].

As we described in Section 4, resveratrol possesses anti-inflammatory effects through various pathways. Chen et al. [103] concluded in their study that resveratrol acts through inhibition of the expression of two enzymes induced by dioxin (CYP1A1 and CYP 1B1) via the AhR pathway. Another possible mechanism involving this natural compound is described by Casper et al., who found that resveratrol, in the presence of TCCD competes with AhR and inhibits CYP1A1 expression, resulting in anti-inflammatory activity [80].

Research of Yavuz S. et al. [104] was carried out to demonstrate the efficiency of resveratrol as a therapeutical agent in the treatment of endometriosis. The study included surgically induced lesions of endometriosis in 24 female rats. Four weeks after the procedure, the injuries were measured in three groups of study: control group, low resveratrol dose (1 mg/kg/day), and high resveratrol dose (10 mg/kg/day). Resveratrol was administered intraperitoneal over seven days, and at the end of the period, a laparotomy was completed for the purpose of observing the volume of endometriotic lesions and serum/tissue levels of antioxidant enzymes also detected. Their study indicated that resveratrol significantly reduced the volume of endometriotic lesions. Histological scores were also decreased in the treated groups compared. Therefore, resveratrol is a phytochemical with potential ameliorative effects in endometriosis, probably due to its anti-oxidative potency.

Another experimental endometriosis model was used in a prospective study of Tekin et al. [105]. The authors aimed to compare the biological activity of resveratrol in patients affected by endometriosis with the effects of leuprolide acetate. Endometriosis experimental pattern was surgically induced in thirty-three female rats, the cohort was divided into the following groups: group 1 (30 mg/kg resveratrol i.m. for 14 days), group 2 (1 mg/kg leuprolide acetate s.c. single dose), group 3 (resveratrol and leuprolide acetate), and group 4 was the control group, with no medication. The treatment was carried out for two weeks, and after administration, the lesions size, histopathology, immunoreactivity to matrix metalloproteinase-2, matrix metalloproteinase-9, and the vascular endothelial growth factor were evaluated. Peritoneal fluid levels of interleukin-6, interleukin-8, and TNF-α were also studied.

Authors concluded that resveratrol alone might be an efficient alternative to leuprolide acetate in the treatment of this pathology. Moreover, their combination decreased the activity of each therapeutical agent, mostly the anti-inflammatory and anti-angiogenic effects. Plasmatic and peritoneal levels of interleukin-6, interleukin-8, and TNF-α were reduced in the group that received only one therapeutical agent (1 and 2), and the volume of implant lesions was also significantly reduced. The study of Amaya et al. summarized these effects of resveratrol, pointing out that low concentrations of resveratrol associated to E2 possess a high estrogenic activity, suggesting that this compound can be considered as a new approach in the treatment of this disease [106].

Sex steroids are used in the treatment of endometriosis and resveratrol, at high doses, has been demonstrated to decrease the proliferation of human endometrial cells through estrogen receptor 1 (ESR 1) [80]. Another study used ovariectomized immunodeficient RAG-2-γ(c) mice with implanted human endometrial cells. Amaya S. et al. administered a one-month treatment with subcutaneous pellets of estradiol, estradiol and progesterone, and estradiol and resveratrol (6, 30, or 60 mg). They concluded that resveratrol functions in low doses as an estrogen agonist and in high doses as an estrogen antagonist.

Another study of Cenksoy et al. investigated the effect of resveratrol as an anti-angiogenic and anti-inflammatory agent in in vivo research on mice with induced endometriosis [26]. They surgically induced endometrial implants in 24 female rats and after the endometriosis foci had been confirmed, they divided the rats into the following groups: a first group that received resveratrol, the second group that received leuprolide acetate, and the control group. The treatment was administered for 21 days, and at the end of the administration, the authors evaluated the volume and histopathology of lesions. Vascular endothelial growth factor and monocyte chemoattractant protein one measurement in peritoneal samples and blood samples were performed.

The mean areas and volumes of the implants decreased after treatment with both resveratrol and leuprolide acetate. Serum and peritoneal levels of MCP-1 and VEGF also appeared to be significantly lower in both groups, so the effectiveness of resveratrol is comparable with that of leuprolide acetate, a well-known therapeutical agent used for endometriosis regression.

A study in vivo and in vitro from 2011 regarding the effects of resveratrol on endometriosis [25] reported that this compound increases cell death and decreases the proliferation of endometriosis lesions, inhibiting the development of this disease. Another in vivo research on rats demonstrated that after experimentally inducing endometriosis in the subjects, and after treatment with resveratrol, the implant sizes decreased, and also the levels of VEGF and MCP-1 from peritoneal fluid. VEGF expression was also suppressed in the endometriosis tissues after treatment [107]. By inhibiting VEGF expression and synthesis, resveratrol acts as an anti-angiogenic compound while the inhibition of synthesis, receptor activity secretion, and chemotactic activity of MCP-1 have anti-inflammatory effects. The same results were identified by Cenksoy et al., who continued their research with the administration of GnRHa, pointing out that resveratrol had the same impact as leuprolide acetate. Resveratrol acts like synthetic estrogens, binding and activating the estrogen receptors [108].

A study of Taguchi et al. [109] demonstrated that resveratrol alone could reduce significantly surviving mRNA expression, but did not induce apoptosis in human endometriotic stromal cells. Also, pre-treatment with resveratrol in the case of endometriosis significantly enhanced TNF-α-related-apoptosis-inducing ligand (TRAIL), known as a pro-apoptotic molecule.

### 5.2. Clinical Studies

Over time, the use of complementary and alternative medicine have been widely studied for the treatment of endometriosis [110] and many plant-based products, including resveratrol, have been reported to exhibit efficacy against this disease. Although the results of preclinical studies have been favorable and have revealed the effectiveness of resveratrol in the treatment of this disease, clinical trials using resveratrol have been limited. The hypothesis of most of the clinical trials involving resveratrol administration was that the combination of oral contraceptives with naturally occurring aromatase inhibitors might show promise for the treatment of endometriosis.

Resveratrol can potentiate the actions of oral contraceptives in the treatment of endometriosis-related symptoms (such as dysmenorrhea). The mechanism of action, in this case, consists of decreasing the expression of cyclooxygenase-2 and aromatase expression [81].

The suppression of aromatase and Cox-2 expression in the endometrium is a necessary premise for the control of chronic pelvic pain. Besides the suppression of aromatase, resveratrol possesses the ability to block SIRT 1 and transform growth factor-beta genes. Synthetic aromatase inhibitors do not share this characteristic [111]. Although the real mechanism of the anti-inflammatory effect of resveratrol is not fully known, it seems to include the inhibition of NF-kB activation and its translocation to cell nuclei. There, it stimulates the transcription of several genes connected to the inflammatory cascade [112].

Because the excess of estrogen in endometriosis takes place in lesions as a consequence of the expression of the aromatase p450 enzyme, progesterone resistance may develop as a consequence of this hyperestrogenic milieu. Therefore aromatase expression may persist into the endometrium of patients using oral contraceptives during the first months of treatment and pelvic pain and bleeding may continue despite the treatment. [113]. The breakthrough that bleeding in oral contraceptive users was associated with Cox-2 and nuclear factor kappa beta activation, thereby suggested that the recommencement of inflammation plays a significant role in the resumption of uterine bleeding and pain.

In this respect, the anti-inflammatory effect of resveratrol will contribute towards decreasing the pain associated with endometriosis, potentiating the therapeutic impact of drospirenone and rendering the patients pain-free [114,115].

Maia H. Jr et al. [116] investigated 12 patients with endometriosis-associated dysmenorrhea, who failed positive results after administration of oral contraceptive, containing drospirenone + ethinylestradiol. They added 30 mg of resveratrol to the standard hormone therapy and concluded that the pain scores significantly reduced after two months of treatment. A separate study of the same authors included 42 women with endometriosis submitted to laparoscopy and hysteroscopy, where they investigated aromatase and cyclooxygenase-2 expression from endometrial tissue of these patients. Sixteen patients used before hospital admission oral contraceptives alone and 26 received oral contraceptives and resveratrol combined. The authors concluded that the inhibition of aromatase and COX-2 was increased in the group with combined therapy.

Mendes da Silva et al. [117] also conducted a randomized clinical trial to observe the effectiveness of resveratrol in the management of endometriosis, associated with monophasic contraceptive pills. They included in the study 44 women aged 20 to 50, who randomly received two pills for 42 days: one tablet was an oral contraceptive and the other 40 mg of resveratrol or placebo pills. After the end of the study, the authors concluded that resveratrol did not prove any additional effects to placebo for the treatment of endometriosis-related symptoms because the differences between median pain scores in the two groups were insignificant.

NF-kB is one of the significant transcription agents involved in the inflammatory pathway of this disease. Another factor that seems to play an essential role in inflammation is an NAD^+^ dependent histone deacetylase, SIRT1. It was discovered that this factor is also involved in carcinogenesis [118].

To observe the expression of sirtuin 1 in this chronic pathology, Taguchi et al. [119] obtained endometriotic stromal cells and exposed them to resveratrol and sirtinol, which are an activator and an inhibitor of sirtinol, respectively. Immunohistochemistry and RT-PCR examined the eutopic endometrial cells, and this study concluded that sirtuin one was identified both in endometriotic stromal and in not affected cells. After the exposure to resveratrol, the authors found that it decreased tumor necrosis factor-α-induced, interleukin-8 release and SIRT1, and increased interleukin-8 release. Therefore, the contrary actions of resveratrol and sirtinol proved that interleukin-8 release is modulated through sirtuin 1. Therefore resveratrol can be used in endometriosis to ameliorate chronic inflammation.

Simvastatin is an inhibitor of 3-hydroxy-3-methylglutaryl-coenzyme A reductase (HMGCR) activity, with intrinsic antioxidant activity [120] that is used in the treatment of endometriosis. In this case, the mechanism of resveratrol does not involve anti-inflammatory pathways. Resveratrol possesses the ability to inhibit HMGCR mRNA expression, whereas both resveratrol and simvastatin inhibit enzymatic activity. HMGCR represent a rate-limiting step of the mevalonate pathway that includes the isoprenoids FPP and GGPP. These isoprenoids are necessary for the isoprenylation of proteins involved in apoptosis, cell proliferation, adhesiveness, and maintenance of cellular functions. Different products of the mevalonate pathway exert negative feedback on HMGCR expression [115]. It is worth mentioning that the effects of resveratrol on the enzymatic activity of HMCGR are independent of the effects on *HMCGR* expression.

So, the addition of resveratrol may potentiate the effect of simvastatin when it is used as a therapeutic agent against endometriosis, through a mechanism that does not involve an anti-inflammatory effect.

To investigate the interactions between simvastatin and resveratrol, focusing on cholesterol biosynthesis and protein activity in cultures of human endometrial stromal cells (HES), Villanueva et al. [121] obtained HES from healthy volunteers. Then, they measured the conversion of acetate to cholesterol and quantified HMGR mRNA transcripts, protein expression, and enzyme activity, by measuring the conversion of 3-hydroxy-3-methyl-glutaryl-coenzyme A to mevalonic acid lactone in HES cells.

The results of this study indicated that resveratrol potentiated the inhibitory effects of simvastatin on cholesterol biosynthesis and the activity of the HMGCR enzyme. It also inhibited the stimulatory effects of statin on protein expression and HMGCR mRNA transcription. Therefore, the combination of resveratrol and Simvastatin may be potentially useful in the development of new management of endometriosis.

Regarding the mechanisms of action of resveratrol, this phytochemical is known to have an anti-inflammatory, anti-angiogenic effect and also to induce apoptosis in various cell types, but its pro-apoptotic role on human endometrial cells remains uncertain.

Given all the presented studies, resveratrol is a promising agent against endometriosis. It has been shown to suppress the expression of various inflammatory biomarkers (TNF-α, COX-2), to activate various transcription factors (NF-kB, PPAR-gamma), and to induce antioxidant enzymes (catalase, superoxide dismutase) [122] and thus it holds promise as a natural therapeutical agent but further studies are necessary in order to establish the doses for human administration.

## 6. Conclusions and Future Perspectives

Endometriosis is a benign gynecological disorder that affects women in the reproductive age worldwide and is characterized mainly by chronic abdominal pain and infertility. Even the pathophysiologic mechanisms of endometriosis are not completely known; chronic inflammation is considered one of the pathways responsible for endometriosis development.

Natural polyphenols are bioactive compounds with multiple beneficial properties that provide new therapeutical perspectives against endometriotic lesions. Polyphenols are known to possess anti-carcinogenic, anti-angiogenic, anti-inflammatory, proapoptotic and anti-oxidative effects. Recently, the anti-inflammatory potential of natural dietary compounds has raised interest for researchers because it might be used in the treatment of endometriosis.

In this article, we summarized some of the epidemiological and clinical research that supports the beneficial effect of resveratrol in endometriosis. Moreover, resveratrol demonstrated its efficiency either alone or associated with other classical therapeutically agents used in endometriosis treatment such as leuprolide acetate or statins.

Knowledge of the precise and more profound mechanisms of how resveratrol can reduce endometriotic lesions is required, and further studies on this topic are crucial. Overall, the role of resveratrol in reducing the volume of endometriotic lesions and chronic abdominal pain is a proven fact.

## Figures and Tables

**Table 1:** Preclinical studies regarding the effects of resveratrol in endometriosis.

Author	Study Design	Number of Cases	Treatment Regimen/Study Design	Follow-Up	Results
**Yavuz et al. [104]**	Prospective study, surgically induced lesions of endometriosis	24 female rats	*Control group*—no treatment *Group 1*—**1 mg/kg/day** of resveratrol, injected intraperitoneally *Group 2*—**10 mg/kg/day** of resveratrol, injected intraperitoneally	7 days	- Endometriotic implants volume and proliferating cell nuclear antigen expression levels were significantly reduced in treated groups. - Also, the increased activity of superoxide dismutase and glutathione peroxidase in serum and tissue of treated rats was detected.
**Tekin et al. [105]**	Prospective study, surgically induced lesions of endometriosis	33 female rats	*Control group*—no treatment *Group 1*—**30 mg/kg** resveratrol i.m for 14 days *Group 2*—**1 mg/kg** leuprolide acetate s.c. single dose *Group 3*—**30 mg/kg** resveratrol i.m. for 14 days and one single dose of **1 mg/kg** leuprolide acetate s.c.	14 days	- The volume of endometriotic implants and the histopathological grade were significantly reduced in treated groups. - Immunoreactivity to MMP2, MMP9, and VEGF was decreased - Comparing the levels of IL-6, IL-8, and TNF-α in plasma and peritoneal fluid, they were significantly reduced in group 1 and 2 compared to group 3 and the control group
**Amaya et al. [106]**	Prospective study, ovariectomized immunodeficient RAG-2-γ(c) mice with human endometrial tissue implanted	N	*Group 1*—Subcutaneous pellets of E2 *Group 2*—Subcutaneous pellets of E2 plus progesterone (P4) *Group 3*—Subcutaneous pellets E2 plus resveratrol (6, 30, or 60 mg) injected intraperitoneally	30 days	- After the administration of treatments, immunohistochemical expression of ESR1, Ki67, reverse transcriptase polymerase chain reaction of AhR, CYP1A1, and CYP1B1 were analyzed - Decreased expression of ESR1 and proliferative activity (Ki67) was exhibited with 60 mg of resveratrol.
**Cenksoy et al. [26]**	Prospective study, surgically induced lesions of endometriosis	24 female Wistar–Albino rats	*Group 1*—resveratrol (7) *Group 2*—leuprolide acetate (8) *Group 3*—control group (7)	21 days	- The mean areas of endometriotic implants were reduced after treatment in both group 1 and group 2. - Histopathological scores of the VEGF scores of endometriotic implants and peritoneal fluid levels of VEGF and MCP-1 were decreased in group 1 and 2
**Taguchi et al. [109]**	Prospective study	Endometriotic tissues collected during surgeries from ovarian endometriosis affected women	Human endometriotic cells were cultured and pretreated with resveratrol in vitro. Then, the cells were incubated with TNF-α-related-apoptosis-inducing ligand		- Resveratrol is not able to induce apoptosis in human endometriotic stromal cells alone - it significantly decreases surviving mRNA expression - enhances TRAIL-induced apoptosis.

**Table 2:** Clinical studies regarding the effects of resveratrol in endometriosis.

Maia Jr. et al. [116]	Prospective Study with Two Arms	Experiment 1: - 12 Patients Treated with Drospirenone Ethinylestradiol 3 mg/30 μg for 6 Months	Experiment 1: All the Patients were Switched to a Combination of Drospirenone/Ethinylestradiol /Resveratrol (a Dose of 30 mg/Day)	Experiment 1: 6 Months	Experiment 1: - Decreased Pain Scores after 2 Months of Treatment - 82% of Patients Reported Complete Resolution of Dysmenorrhea and Pelvic Pain
		**Experiment 2**: - 42 patients surgical treatment. - 16 treatment with oral contraceptives - 26 combination with resveratrol	**Experiment 2:** - 16 patients used drospirenone/ethinylestradiol for at least 2 months before surgery. - 26 patients drospirenone/ethinylestradiol associated with 30 mg of resveratrol.		**Experiment 2:** - Expression of both aromatase and cyclooxygenase-2 was reduced in the eutopic endometrium of patients using drospirenone/ ethinylestradiol associated with 30 mg of resveratrol, compared with the endometrium of patients using oral contraceptives alone.
**Mendes da Silva et al. [117]**	Prospective study, double-blinded trial	44 women with a laparoscopic diagnosis of endometriosis	The patients were randomized to receive oral contraceptives or 40 mg resveratrol/day or placebo pills.	42 days	- In the placebo group, mean pain scores were 5.4 before treatment and in resveratrol group were 5.7. After the procedure, the mean pain scores registered were 3.9 in the placebo group and 3.2 in the resveratrol group.
**Villanueva et al. [121]**	Prospective study	Endometrial tissue was obtained from 8 patients, undergoing surgeries or healthy volunteers	Cholesterol biosynthesis by human endometrial cells was assessed in vitro by measuring the conversion of [^14^C] acetate to [^14^C] cholesterol in the presence of resveratrol (30–100 μM), simvastatin (0.1–10 μM), or resveratrol 30 μM + simvastatin 0.1 μM		- Resveratrol inhibited cholesterol biosynthesis, enzyme activity, and HMGCR mRNA and potentiated the inhibitory effects of simvastatin on cholesterol biosynthesis and HMGCR enzyme activity.

## Abbreviations

AA	arachidonic acid
AhR	aryl hydrocarbon receptor
AKT	protein kinase B
AP	activator protein
ATF	activating transcription factor
c-Jun	protein encoded by the JUN gene
CO2	carbon dioxide
CoA	coenzyme A
COX	cyclo-oxygenase
DNA	deoxyribonucleic acid
E2	estradiol
E2F1	transcription factor encoded by the E2F1 gene
ERK-1	extracellular signal-regulated kinase 1
ESR 1	estrogen receptor 1
FOS	protein family of transcription factors
FoxP3+	regulatory T (Treg) cells
FPP	farnesylpyrophosphate
GGPP	geranylgeranylpyrophosphate
GM-CSF	granulocyte macrophage colony-stimulating factor
GnRH	gonadotropin-releasing hormone
H2O2	hydrogen peroxide
HES	human endometrial stromal cells
HIF-1	hypoxia-inducible factor 1α
HMGCR	3-hydroxy-3-methylglutaryl-coenzyme A reductase
IL	interleukin
IL-1β	interleukin 1β
JNK	c-Jun N-terminal kinases
LPS	lipopolysaccharide
LTB4	leukotriene B4
MAF	transcription factor
MAPK	mitogen-activated protein kinase
MAPK1/3; ERK1/2	mitogen-activated protein kinase 1/3
MAPK14	mitogen-Activated Protein Kinase 14
MCP-1	monocyte chemoattractant protein 1
MIF	macrophage inhibitory factor
MMP	metalloproteinase
mRNA	messenger ribonucleic acid
NAD	nicotinamide adenine dinucleotide
NFAT	nuclear factor of activated T-cells
NF-kB	nuclear factor-kB
NO	nitric oxide
O_2_	oxygen
PG	prostaglandin
PGE2	prostaglandin G2
PPAR	peroxisome proliferator-activated receptors
PGF2α	prostaglandin F2α
PGG2	prostaglandin G2
PGH2	prostaglandin H2
PGI2	prostaglandin I2
RPS6KB2	ribosomal protein S6 kinase beta-2
RT-PCR	reverse transcription polymerase chain reaction
SIRT1	sirtuin 1
STATs	signal transducer and activator of transcription protein family
STAT3	signal transducer and activator of transcription 3
Th17	lymphocytes T helper 17
TNF-α	tumor necrosis factor
TRAIL	TNF-α-related-apoptosis-inducing ligand
UV	ultraviolet
VEGF	vascular endothelial growth factor
VEGFC	vascular endothelial growth factor C
VEGFR	vascular endothelial growth factor receptor
AA	arachidonic acid
AhR	aryl hydrocarbon receptor
AKT	protein kinase B
